# An international questionnaire highlights and supports the case for including girls in Creatine Transporter Deficiency research

**DOI:** 10.3389/fnins.2025.1620586

**Published:** 2025-07-07

**Authors:** Carole Chehowah, Audrey Mittelman, Vincent des Portes

**Affiliations:** ^1^Association Xtraordinaire, Paris, France; ^2^Creatine Transporter Deficiency Commission, Paris, France; ^3^Department of Pediatric Neurology, Hôpital Femme Mère Enfant, Bron, France

**Keywords:** X-linked, Creatine Transporter Deficiency, SLC6A8, gender disparity, patient advocacy organization, girls with CTD

## Abstract

Over the last 15 years, significant progress has been made for Creatine Transporter Deficiency (CTD) patients, with increased awareness and visibility, better diagnosis, and improved care. Research projects have paved the way for clinical trials on the horizon. However, girls with CTD have been overlooked. Because they are considered a negligible cohort with less serious symptoms compared to males, girls with rare X-linked disorders have never been a priority for diagnosis and research. This results in underdiagnosis, systematic exclusion from clinical studies, and a considerable impact on the development of female patients. As a patient association, Xtraordinaire aims to counter these beliefs and to show that females deserve as much attention as males. Our first initiative identified girls with CTD within our community and led to the development of an international questionnaire to collect more specific data in this population. Of the 22 families who completed the questionnaire, the delay between symptom onset (mean age 1.8 years) and diagnosis (mean age 11.8 years) highlighted the difficulty of diagnosis for girls, often given several wavering diagnoses before reaching a CTD diagnosis. Almost half of families (47%) had difficulties securing a specialist appointment. Our questionnaire emphasized that girls with CTD have identical symptoms to males and similar delays in development milestones. These data have generated interest, and researchers have started to include girls in their studies. We strongly believe that upcoming studies on females will enrich our knowledge, further our understanding of CTD, help better diagnose girls, and develop adapted treatments.

## 1 Introduction

Xtraordinaire is a French non-profit organization, founded by parents in 2006, for families and patients with rare X-linked syndromes associated with neurodevelopmental disorders, such as Creatine Transporter Deficiency (CTD). Xtraordinaire is a community dedicated to improving the lives of patients and their families, educating and building awareness, advocating to shorten the path to diagnosis, and promoting, encouraging, and funding research programs. Over the years, our initiatives have created a strong network and have strengthened partnerships between researchers, physicians, and patients.

Xtraordinaire’s commission on CTD (SLC6A8) was also created in 2006 with only six CTD families and now represents almost 90% of the French CTD families (103 families with a known diagnosis). Since its inception, the CTD commission has worked to create family networks, improve diagnosis, visibility, and encourage research. We have also organized several international CTD symposia attended by families, researchers, and experts (Chehowah, 2025). After more than 15 years, our efforts are paying off, with a drug candidate being developed in France, and clinical trials around the corner (Disdier et al., 2025). The CTD commission is also in contact with international CTD families and has been partnering with the US Association for Creatine Deficiencies (ACD) since 2016.

CTD (SLC6A8/CTD), an X-linked genetic disorder caused by a variant in the SLC6A8 gene located on the X chromosome, is one of three cerebral creatine deficiency syndromes (CCDS) (Fernandes-Pires and Braissant, 2022). It is a very rare genetic mutation on the X chromosome that impairs the ability of the transporter to bring creatine into brain cells (Fernandes-Pires and Braissant, 2022). It mainly affects males but around 10% of females are also affected (Curie et al., 2024). Approximately 30% of pathogenic variants have been reported to occur *de novo*, while the rest are inherited from the mother (DesRoches et al., 2015). The severity of CTD can vary from patient to patient, but it causes neurodevelopmental disorders, such as intellectual disability, speech delays, learning disabilities, autism spectrum disorders (ASD), motor skill difficulties, movement disorders, hypotonia, behavior disorders, and epilepsy/seizures (Curie et al., 2024).

To date, numerous studies on CTD have been published, and clinical trials are in the pipeline (Disdier et al., 2025; Fernandes-Pires and Braissant, 2022). However, the vast majority have been carried out in male patients, in male animal models, or on male fibroblasts (Brandabur et al., 2024; Broca-Brisson et al., 2024; Ghirardini et al., 2021; Thurm et al., 2016). In the case of CTD, the severity of symptoms varies with each unique variant (Gechijian et al., 2024), which is true for both male and female patients, according to our experience. Even if girls only account for around 10% of the small number of patients diagnosed with CTD worldwide (Curie et al., 2024; Fernandes-Pires and Braissant, 2022), we feel that it is essential for girls to be included in clinical studies.

The complexity of CTD due to its many variants (Ferrada et al., 2024) combined with the persistent and possibly mistaken belief that women and girls have milder to no symptoms have a real impact on diagnosis (Mejdahl Nielsen et al., 2023). Another difficulty in accessing diagnosis for girls is that the urine and blood creatine screenings typically used in males to identify an increase in the creatine/creatinine ratio is not reliable in heterozygous females (Mejdahl Nielsen et al., 2023). The path to diagnosis is, therefore, complicated, as it requires brain magnetic resonance spectroscopy and DNA sequencing, which are more costly than urine analysis and require access to specialists (Ghirardini et al., 2021). This might in itself explain why the proportion of girls diagnosed with CTD is low compared with males (Mejdahl Nielsen et al., 2023; Sharer et al., 2017).

Because X-linked disorders such as CTD are genetic conditions often perceived as primarily affecting males, we found that clinical trials for X-linked disorders have historically excluded women (Liu and Mager, 2016; Waltz et al., 2023; Yakerson, 2019), explaining why they are not included in research projects and studies, and resulting in knowledge gaps about how rare diseases present and progress in female patients. Our original search for publications and data mentioning girls with CTD highlighted the gender disparity faced by female CTD patients. As a result, we developed a first questionnaire carried out in 2022 among seven French-speaking families with a CTD daughter, presented at the International Symposium of Cerebral Creatine Deficiency Syndromes in June 2022 (Chehowah, 2022). The average age of CTD diagnosis overall was 8.7 years, but 11.9 years for girls. Our original survey highlighted that, despite lighter symptoms/disorders for some girls, most were diagnosed with light intellectual disability (ID) or ASD. Families encountered difficulties securing appointments with specialists and were not encouraged to pursue a genetic diagnosis. The severity of symptoms/disorders was similar regardless of gender and ranged from mild to severe. The most disabling severe symptoms, such as ID, aggressiveness, epilepsy, and ASD were as common in girls than in boys. Given the interest garnered by the International Symposium of Cerebral Creatine Deficiency Syndromes presentations on girls in 2022 and 2023 (Chehowah, 2022; Symposium International DTC (SLC6A8/CTD) Paris Sept 29/30, 2023), and the requests from non-French families, we undertook a second, more detailed questionnaire aimed at international families, the results of which are presented below.

## 2 Materials and methods

### 2.1 Questionnaire

The study’s instrument was a self-administered 33-item questionnaire (Supplementary Material). The questionnaire was elaborated in French by Xtraordinaire with the help of Prof. Vincent des Portes (Head of Pediatric Neurology, Hôpital Femme Mère Enfant, Bron, France), whose expertise in rare syndromes with neurodevelopmental disorders combined with the author’s experience as the mother of a daughter with CTD and as an advocate for CTD patient associations allowed the design of questions to suit the specific characteristics of the syndrome, based on patients’ development, symptoms, and needs. We also made sure that it would not be too time-consuming for the families, with the objective to address all the relevant criteria and information that could be used in future studies and trials.

Content validity testing was performed by sending the questionnaire to a panel of three experts to validate the importance and intelligibility of the questionnaire before the final version was distributed. Finally, it was translated into English, Italian, and Dutch by members of respective CTD patient advocacy organizations.

The questionnaire was divided into sections on the characteristics, family, symptom, and diagnosis history as well as on the current symptoms of girls with CTD, including treatment strategies. It included sections on the ages at which they reached developmental milestones, such as sitting or walking without support, and language development. Further, the questionnaire asked about education and activities of daily living and acquisition of autonomy, motor skills, learning skills, or socialization. Finally, the survey also tracked behavior disorders and symptom evolution in the past year, enquired on which situations can cause stress, on the presence of eating or gastrointestinal disorders, or any other health problems/medical conditions (Supplementary Material).

### 2.2 Data collection

The questionnaire was launched in early 2023 and was sent by email through our network and with the help of our contacts in other countries (ACD in the United States, local contacts in Italy, Spain, Holland, Switzerland, Germany, Belgium, and Australia). Participation was anonymous and the questionnaire was GDPR compliant.

In June 2023, our networks internationally knew of 27 families with a CTD girl. The questionnaire was completed by 22 families, representing 80% of the girls diagnosed with CTD. The questionnaires were mainly completed by their mothers, as the majority of girls with CTD did not have the capacity to answer themselves.

### 2.3 Data analysis

Data analysis was carried out by calculating percentages of responses for each question. Given this data evaluation procedure, no power calculation (*a priori* or posteriori) was necessary. The free text responses addressed were analyzed and reviewed. The results of the analyses were interpreted and integrated for both the questionnaire results and the free text fields.

## 3 Questionnaire results of girls with CTD

### 3.1 Diagnosis

Our questionnaire found that the mean age of CTD girls was 18.8 years (min: 3, max: 48), their mean age at first symptom onset was 1.8 years, and that their mean age at diagnosis was 11.8 years. This represents a wide difference in mean age for diagnosis compared with CTD males, who are diagnosed at 4.9 years (Brandabur et al., 2024), despite girls experiencing early and numerous symptoms.

The time elapsed between first symptom onset and the diagnosis underlines the difficulty of diagnosis for girls. Indeed, there were several wavering diagnoses before reaching an accurate CTD diagnosis. Those mainly included psychological problems, ASD, hyperactivity, pervasive developmental disorders, despite numerous proven symptoms such as speech delays (77%), developmental gap with other children (77%), motor skills difficulties (68%), learning delays (59%), epilepsy/seizures (59%), global developmental delays (54%), and gastrointestinal problems (45%). In 47% of cases, families reported difficulties with getting an appointment with a specialist.

### 3.2 Epilepsy/seizures

Thirteen (59%) girls had epilepsy. In 10 cases (76%), multiple types of epileptic fit were reported, including absence or alteration of consciousness for all, convulsive seizures (30%), spasms and hyperkinetic (23%), language alteration (30%), hallucinations (23%), enuresis (23%), agitation/ inconsistent statement (15%), chewing (15%), abdominal pain in one case, and absence or alteration of consciousness (23%). In the Vigilan observational study, which only included CTD boys (*n* = 50), 68% were reported to have a medical history of seizures, and 50% reported hypotonia (Brandabur et al., 2024).

Reflecting the wide range of symptoms, treatments vary from case to case with monotherapy and combination therapy. Treatment was effective for 10 girls, while 3 girls still had seizures despite numerous treatments. Thus, there was no correlation between the type of epileptic fit and treatment effectiveness. Eleven girls experienced side effects associated with anti-epileptic treatments.

### 3.3 Main development milestones

Regardless of the development milestone, questionnaire responses indicated delays in acquisition and development for girls, similar to results reported in boys (Brandabur et al., 2024).

**Figure 1:**
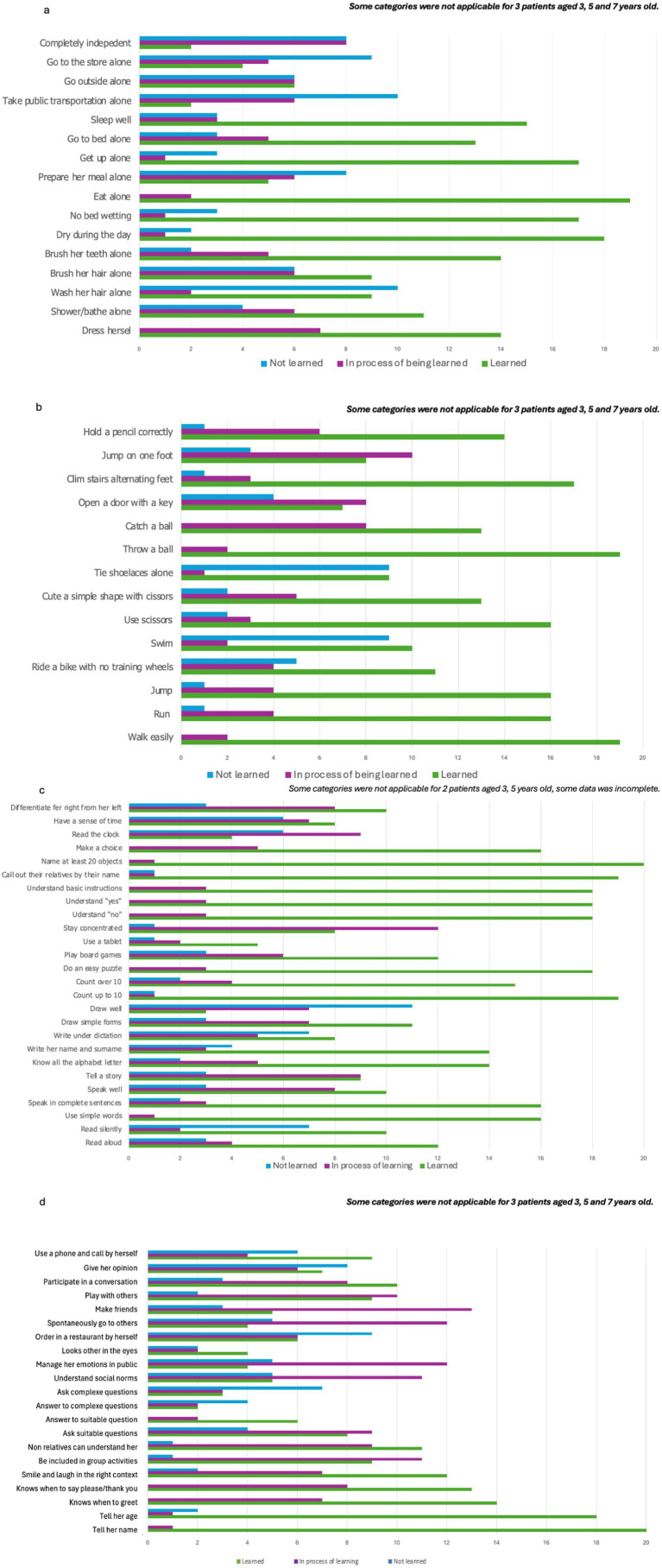
Main development milestones. **(a)** Autonomy, **(b)** motor skills, **(c)** learning skills, **(d)** socialization.

Regarding autonomy, the categories with the least acquired skills were those relating to the outside world and all the difficulties that this entails on a daily basis (Figure 1a). For motor skills, acquisition of most fine motor skills and balance were delayed (Figure 1b). For learning skills, only basic skills were acquired or in acquisition (Figure 1c). For socialization, all categories except “tell her name” showed a real difficulty in socializing (Figure 1d).

**Figure 2:**
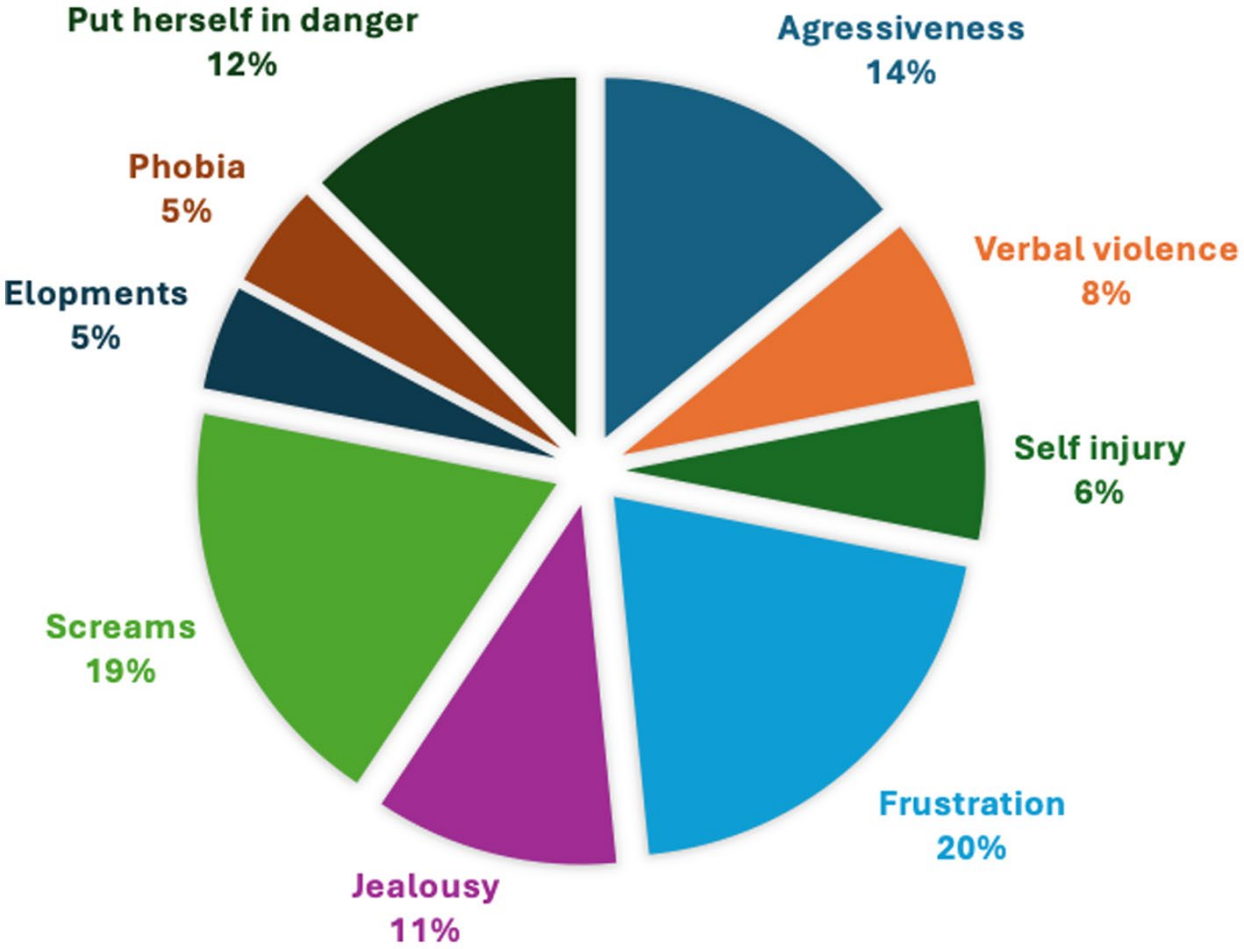
Behavioral issues.

Behavioral issues were reported for 72% of the girls, including frustration, screams, self-injury, aggressiveness, no sense of danger, jealousy, phobia, and elopement (Figure 2). In comparison, the Vigilan observational study in CTD boys reported aggressive behavior (46%), self-injurious behavior (40%), and anxiety disorder (20%) (Brandabur et al., 2024).

### 3.4 Key development milestones

Girls with CTD exhibited notable delays in reaching key developmental milestones compared to typically developing children (Dosman et al., 2012). Data indicated that only 58.8% of girls were able to sit without support by 9 months of age; all girls in the cohort achieved this skill by 18 months. In children who do not have CTD, sitting without support occurs between 3.8 months and 9.2 months (WHO, n.d.). The acquisition of independent walking was also delayed: just 38.8% of girls were able to walk without help by 18 months, and 100% reached this milestone by 2 years and a half; children without CTD reach the milestone of walking alone between 8.2 months and 17.6 months (WHO, n.d.). Language development was particularly affected: while 80% of girls were able to form simple two- or three-word sentences after the age of 3, this milestone was reached by all girls by the age of 9 years. In comparison, baseline language levels in the Vigilan observational study, which only included CTD boys (mean baseline age 8.8 years) were single words (42%), sentences (32%), babbling (16%), none (4%), and unknown (4%) (Brandabur et al., 2024). According to American Speech Language Association,^1^ children without CTD acquire 1 or 2 words by 12 months and form their first sentence between 12 and 18 months.

These findings underscore a pattern of global developmental delays in girls with CTD, with gross motor skills such as sitting and walking achieved later than in the general population, and expressive language milestones reached much later still (Dosman et al., 2012). The data suggest that, while most girls eventually attain these fundamental skills, the trajectory is significantly protracted, especially for language. This highlights the need for early intervention and tailored support to address the unique developmental challenges faced with CTD and emphasizes the importance of long-term monitoring to optimize functional outcomes.

## 4 Conclusions and future perspectives

In 2022, when we presented the results of the first questionnaire on girls with CTD at the CCDS symposium in Utah (Chehowah, 2022), we were overwhelmed with the reception and interest from the audience, particularly from American families with a CTD daughter. It was a trigger to continue and launch a second questionnaire worldwide. All our presentations whether at conferences, symposia, or more formal meetings, have since included data on female patients with CTD.

In 2023, at the CTD/CCDS international Symposium in Paris, France, we dedicated a full session on girls with CTD for the first time (Symposium International DTC (SLC6A8/CTD) Paris Sept 29/30, 2023). The session consisted of four presentations and a roundtable discussion focused on females affected by X-linked conditions. Taylor Kane, founder of Remember the Girls, opened by sharing her personal journey and the organization’s mission to raise awareness about all females impacted by X-linked syndromes, including both symptomatic and asymptomatic carriers, and to address the guilt often felt by mothers. Jiddeke Van de Kamp discussed the genetic differences between males and females in X-linked CTD. As discussed, the authors presented key findings of a questionnaire about girls with these conditions in France. Professor Olivier Braissant introduced his research on a female animal model for CTD. The session concluded with a roundtable featuring experts who discussed the challenges of diagnosis, the need for more data, and the importance of recognizing and including mothers and sisters in research and support efforts (Symposium International DTC (SLC6A8/CTD) Paris Sept 29/30, 2023).

In addition, the Xtraordinaire CTD commission has been proactive in raising awareness and convincing experts and researchers to include girls in future studies and projects. This has enabled us to make significant progress, with two projects already completed for boys that are now underway for girls, as detailed below.

1. Dr. Aurore Curie, CHU Lyon, France, is opening the CREAT’CRITERIA study to 15 CTD girls, after completing the study on boys (Curie et al., 2024). She is currently recruiting CTD girls to participate in this prospective study designed to determine the most relevant outcome measures to use in future clinical trials. Thus, it is key to also have these data and assessments in girls.
2. Dr. Aloïse Mabondzo, CEA France, is launching a new study “Bridge the Gap Between Preclinical and Clinical Therapy for Creatine Transporter Deficiency for Female Patients Using Human Brain Organoids.” In order to further understand the pathophysiology of CTD patients, Dr. Mabondzo will include heterozygote patients. It is an important step to include female CTD patients in clinical trials, in order to verify the relevance of therapeutic follow-up markers.

Next, we will advocate for companies developing treatments and for health authorities to include girls in clinical trials. Advocating for females with CTD, raising awareness about their symptoms, better understanding their differences, and explaining how to better screen girls with CTD are essential to learn more about this rare disease. All efforts to increase the visibility of girls with CTD have been applauded and encouraged. Our questionnaires and presentations have generated a lot of interest from researchers and experts, and demonstrated that there are plenty of good reasons to improve our knowledge of girls with CTD and to include them in all future studies, projects and clinical trials.

Women were historically excluded from clinical trials, which led to significant gaps in knowledge about drug safety, efficacy, and disease manifestation in women (Liu and Mager, 2016; Waltz et al., 2023; Yakerson, 2019). Policies such as the NIH Revitalization Act and FDA guidelines now mandate the inclusion of women, recognizing that diverse participation leads to more robust and generalizable findings. Ultimately, involving girls and women in clinical trials for X-linked diseases is not only a matter of scientific rigor but also of health equity—empowering all patients to benefit from advances in medical research.

In conclusion, the data collected by Xtraordinaire via our questionnaire confirmed that all the symptoms and neurodevelopmental disorders observed in male patients with CTD are also seen in female patients with CTD, highlighting the importance of including girls in clinical research. Excluding women leads to critical gaps in knowledge that may result in underdiagnosis, undertreatment, or inappropriate dosing for female patients. For a rare disease with so few patients, to exclude girls is to deprive ourselves of more data and knowledge about the disease.

## Data Availability

The original contributions presented in the study are included in the article/Supplementary material, further inquiries can be directed to the corresponding authors.

## Footnotes

^1^ https://www.asha.org/
