# Supplementary material for: An international questionnaire highlights and supports the case for including girls in Creatine Transporter Deficiency research
Source: Front Neurosci. 2025 Jul 7;19:1620586. doi: 10.3389/fnins.2025.1620586 (PMC12277310; doi:10.3389/fnins.2025.1620586)
Supplement: Supplementary file 1 [file Data_Sheet_1.pdf]

XTRAORDINAIRE - CTD GIRLS QUESTIONNAIRE

Participation is anonymous and the questionnaire is GDPR compliant. The data will be compiled and used for the presentation "Girls with Creatine Transporter Deficiency" at the September 2023 Symposium in Paris.

WHO IS FILLING THE QUESTIONNAIRE

1. MOTHER
2. FATHER
3. LEGAL GUARDIAN
4. OTHER

YOUR CTD GIRL - YEAR OF BIRTH

DO YOU HAVE ANOTHER CTD CHILD OR PERSON WITH CTD IN YOUR FAMILY ?

1. YES
2. NO

IF YES (please check all that apply)

IT IS A GIRL

IT IS A BOY

HE/SHE IS YOUNGER THAN YOUR CTD GIRL

YOUR CTD GIRL - AT WHAT AGE DID YOU NOTICE THE FIRST SYMPTOMS ?

IF YOU REMEMBER, AT WHAT AGE DID YOUR DAUGHTER SIT WITHOUT SUPPORT ?

1. 6 MONTHS
2. 7 MONTHS
3. 8 MONTHS
4. 9 MONTHS
5. 10 MONTHS
6. 11 MONTHS
7. 12 MONTHS
8. BETWEEN 12 & 18 MONTHS
9. BETWEEN 18 & 24 MONTHS
10. BETWEEN 2 & 3 YEARS

11. AFTER 3 YEARS

12. NEVER

<u>IF YOU REMEMBER, AT WHAT AGE DID YOUR DAUGHTER START WALKING WITHOUT HELP ?</u>

1. 9 MONTHS
2. 10 MONTHS
3. 11 MONTHS
4. 12 MONTHS
5. 13 MONTHS
6. 14 MONTHS
7. 15 MONTHS
8. 16 MONTHS
9. 17 MONTHS
10. 18 MONTHS
11. 19 MONTHS
12. 20 MONTHS
13. 21 MONTHS
14. 22 MONTHS
15. 23 MONTHS
16. 24 MONTHS
17. BETWEEN 2 & 2 1/2 YEARS
18. BETWEEN 2 1/2 & 3 YEARS
19. BETWEEN 3 & 4 YEARS
20. AFTER 4 YEARS
21. NEVER

<u>IF YOU REMEMBER, AT WHAT AGE DID YOUR DAUGHTER START 2 OR 3 WORD SENTENCES ?</u>

1. 18 MONTHS
2. 2 YEARS
3. BETWEEN 2 & 2 1/2 YEARS
4. BETWEEN 2 1/2 & 3 YEARS

5. BETWEEN 3 & 4 YEARS

6. BETWEEN 4 & 5 YEARS

7. BETWEEN 5 & 6 YEARS

8. BETWEEN 6 & 7 YEARS

9. BETWEEN 7 & 8 YEARS

10. BETWEEN 8 & 9 YEARS

11. BETWEEN 9 & 10 YEARS

12. AFTER 10

13. NEVER

<u>WHAT SYMPTOM(S) DID YOU NOTICE BEFORE DIAGNOSIS ? please check all that apply</u>

NOTHING

GLOBAL DEVELOPMENT DELAY

INTELLECTUAL DEFICIENCY

SPEECH DELAY

LEARNING DELAY

DEVELOPMENT GAP WITH OTHER CHILDREN

EPILEPSY

SEIZURES

CONCENTRATION DIFFICULTIES

AUTISTIC SYNDROM DISORDERS

BEHAVIOURAL DISORDERS

COMMUNICATION DISORDER

AGRESSIVITY / SELF INJURY

GASTROINTESTINAL PROBLEMS

RELATIONSHIP WITH OTHER CHILDREN

RELATIONSHIP WITH ADULTS (EX PARENTS)

SLEEPING DIFFICULTIES

BED WETTING / TOILETING ISSUES

MOTOR SKILLS DIFFICULTIES

ISOLATION

DOESN'T FEEL THE PAIN

WHICH SYMPTOM(S) HAS/HAVE PUSH YOU TO SEE A DOCTOR AND SEEK FOR A DIAGNOSTIC ?

AGE OF CTD DIAGNOSIS

WHO SUGGESTED / PROVIDED / DID THE DIAGNOSIS ?

please check all that apply

GENERAL PRACTITIONER

PEDIATRICIAN

PEDIATRIC NEUROLOGIST

NEUROLOGIST

GENETICIST

PSYCHIATRIST

TEACHER

WHAT TESTS HAVE BEEN PERFORMED ?

please check all that apply

CREATINE LEVEL IN THE BLOOD

CREATINE LEVEL IN URINE

DNA ANALYSIS

MRS to assess the creatine peak in the brain

OTHER

CAN YOU PLEASE SHARE THE DETAILED SLC6A8 DELETION/MUTATION (DNA SEQUENCING RESULTS) ?

IS IT A "DE NOVO" MUTATION* ? not transmitted by the parents

YES

NO

I DON'T KNOW

I AM NOT WILLING TO ANSWER

WAS IT DIFFICULT TO GET AN APPOINTMENT WITH A SPECIALIST ?

1. YES

2. NO

WAS IT DIFFICULT TO GET A DIAGNOSIS ?

please explain

DID YOUR DAUGHTER GET ANOTHER DIAGNOSIS BEFORE CTD ?

please check all that apply

AUTISM

HYPER ACTIVITY

EMOTIONNAL PROBLEMS

PSYCHOLOGICAL PROBLEMS

PERVASIVE DEVELOPMENT DISORDERS

NONE

EPILEPSY : CURRENT FREQUENCY

NOT CONCERNED / NONE

SHE HAS NO MORE SEIZURE

ONCE PER DAY

MORE THAN ONCE PER DAY

ONCE PER WEEK

MORE THAN ONCE PER WEEK

ONCE PER MONTH

MORE THAN ONCE PER MONTH

ONCE PER YEAR

MORE THAN ONCE PER YEAR

EPILEPSY : SYMPTOMS

NOT CONCERNED / NONE

CONVULSIVE SEIZURES

ABSENCE / ALTERATION OF CONSCIOUNESS

STARING EPISODES

EPILEPTIC SPASMS

HYPERKINETIC

ABDOMINAL PAIN

ENURESY

VISUAL HALLUCINATIONS

CHEWING

EPILEPSY : WHAT TREATMENT(S) / MEDICATION(S) HAVE BEEN TRIED ? WHICH ONE(S) ? (if not concerned, write "nc")

EPILEPSY : TREATMENT EFFECTIVENESS

YES

NO

PARTIALLY

MY DAUGHTER IS NOT CONCERNED

EPILEPSY : IF "YES", WHICH TREATMENT(S)/DRUG(S) SHOWED EFFECTIVENESS ?

EPILEPSY : HAS YOUR DAUGHTER TAKEN CREATINE, ARGININE & GLYCINE SUPPLEMENTS ? please check all that apply

NO

YES AND IT HAS HELPED REDUCING THE NUMBER OF SEIZURES

YES BUT IT DIDN'T HAVE ANY EFFECTIVENESS

EDUCATION, SCHOOL, DAYCARE,... ?

please check all that apply

PRESCHOOL

ELEMENTARY SCHOOL

MIDDLE SCHOOL

HIGH SCHOOL

INDIVIDUALIZED EDUCATION PLAN (IEP)

INCLUSION WITH SUPPORT

SPECIAL / DISABILITY EDUCATION CLASSROOMS

AUTISTIC SUPPORT CLASSROOM

HOMESCHOOLING

SPECIAL NEEDS SCHOOL

UNSCHOOLING

GROUP HOME

APPRENTICESHIP

NONE BY LACK OF PLACE

NONE BY CHOICE

WORKING IN A COMPANY/BUSINESS

HOW IS YOUR DAUGHTER TODAY ?

AUTONOMY “LEARNED” “IN PROCESS OF BEING LEARNED” “NOT LEARNED”

DRESS HERSELF

SHOWER/BATHE ALONE

WASH HER HAIR ALONE

BRUSH HER HAIR ALONE

BRUSH HER TEETH ALONE

NO BED WETTING

DRY DURING THE DAY

EAT ALONE

PREPARE HER MEAL ALONE

GET UP ALONE

GO TO BED ALONE

SLEEP WELL

TAKE PUBLIC TRANSPORTATION ALONE

GO OUTSIDE ALONE

GO TO STORE ALONE

COMPLETELY INDEPENDENT

<u>MOTOR SKILLS “LEARNED” “IN PROCESS OF BEING LEARNED” “NOT LEARNED”</u>

WALK EASILY

RUN

JUMP

RIDE A BIKE WITH NO TRAINING WHEELS

SWIM

USE SCISSORS

CUT A SIMPLE SHAPE WITH CISSORS

TIE SHOELACES ALONE

THROW A BALL

CATCH A BALL

OPEN A DOOR WITH A KEY

CLIMB STAIRS ALTERNATING FEET

JUMP ON ONE FOOT

HOLD A PENCIL CORRECTLY

<u>LEARNING SKILLS “LEARNED” “IN PROCESS OF BEING LEARNED” “NOT LEARNED”</u>

READ ALOUD

READ SILENTLY

USE SIMPLE WORDS

SPEAK IN COMPLETE SENTENCES

SPEAK WELL

TELL A STORY

KNOW ALL THE ALPHABET LETTERS

WRITE HER NAME AND SURNAME

WRITE UNDER DICTATION

DRAW SIMPLE FORMS

DRAW WELL

COUNT UP TO 10

COUNT OVER 10

DO AN EASY PUZZLE

PLAY BOARD GAMES

STAY CONCENTRATED

UNDERSTAND "YES"

UNDERSTAND "NO"

UNDERSTAND BASIC INSTRUCTIONS

CALL OUT THEIR RELATIVES BY THEIR NAMES

NAME AT LEAST 20 OBJECTS

MAKE A CHOICE

READ THE CLOCK

HAVE A SENSE OF TIME

DIFFERENTIATE HER RIGHT FROM HER LEFT

SOCIALIZATION “LEARNED” “IN PROCESS OF BEING LEARNED” “NOT LEARNED”

TELL HER NAME

TELL HER AGE

KNOWS WHEN TO GREET

KNOWS WHEN TO SAY PLEASE/THANK YOU

SMILE AND LAUGH IN THE RIGHT CONTEXT

BE INCLUDED IN GROUP ACTIVITIES

NON RELATIVES CAN UNDERSTAND HER

ASK SUITABLE QUESTIONS

ASK COMPLEX QUESTIONS

UNDERSTAND SOCIAL NORMS

MANAGE HER EMOTIONS IN PUBLIC

ORDER IN A RESTAURANT BY HERSELF

SPONTANEOUSLY GO TO OTHERS

MAKE FRIENDS

PLAY WITH OTHERS

PARTICIPATE IN A CONVERSATION

GIVE HER OPINION

USE A PHONE AND CALL BY HERSELF

<u>BEHAVIOUR DISORDERS</u>

(please check all that apply)

AGRESSIVENESS

SELF INJURY

FRUSTRATION

JEALOUSY

SCREAMS

ELOPEMENTS

PUT HERSELF IN DANGER

NOT CONCERNED

<u>HOW HAVE HER SYMPTOMS EVOLVED IN THE PAST YEAR ?</u>

1. HAVE WORSENED

2. HAVE IMPROVED

3. HAVEN'T CHANGED

<u>WHAT SITUATIONS CAN CAUSE STRESS</u>

(nc if not concerned)

<u>DOES SHE HAVE ANY EATING OR GASTROINTESTINAL DISORDERS ?</u>

(nc if not concerned)

<u>DOES SHE HAVE ANY OTHER HEALTH PROBLEMS / MEDICAL CONDITIONS</u> (heart, blood,...)?

please explain

.
